# Identification of Contactin-1 as a Potential Biomarker and Therapeutic Target in Neuroblastoma

**DOI:** 10.3390/biomedicines12112606

**Published:** 2024-11-14

**Authors:** Christa N. Grant, Carson A. Wills, Xiaoming Liu, Longgui Chen, Zhenqiu Liu, Hong-Gang Wang

**Affiliations:** 1 Division of Pediatric Surgery, Department of Surgery, The Pennsylvania State University College of Medicine, 500 University Drive, Hershey, PA 17033, USA; 2 Division of Pediatric Hematology and Oncology, Department of Pediatrics, The Pennsylvania State University College of Medicine, 500 University Drive, Hershey, PA 17033, USA; carson.wills@pennmedicine.upenn.edu (C.A.W.); xiaoming.liu1116@gmail.com (X.L.); lchen1@pennstatehealth.psu.edu (L.C.); 3 Department of Public Health, The Pennsylvania State University College of Medicine, 500 University Drive, Hershey, PA 17033, USA; liuzx0123@gmail.com

**Keywords:** neuroblastoma, CNTN1, MYCN, biomarker, prognosis

## Abstract

Background: Neuroblastoma is a common pediatric solid tumor with poor outcomes in high-risk patients. The identification of new therapeutic biomarkers is critical for the treatment of disease. Methods: An analysis of large publicly available datasets of tumor gene expression was performed. In vivo studies were performed to elucidate the role of contactin-1 (CNTN1) in tumor progression. Results: Expression of the glycoprotein CNTN1 is elevated in neuroblastoma compared to other tumor types. CNTN1 expression is higher in stage 1 and non-MYCN-amplified tumors, compared to more aggressive stage 4 and MYCN-amplified tumors. Moreover, high CNTN1 expression is associated with increased overall survival in neuroblastoma patients. In vivo studies demonstrate reduced metastasis in mice xenografted with CNTN1 knockout tumors compared to wildtype. Conclusions: The results of this study suggest that CNTN1 is a potential biomarker and therapeutic target in neuroblastoma. Further investigation of CNTN1 could have significant clinical implications for improving neuroblastoma treatment.

## 1. Introduction

Neuroblastoma is a common extracranial pediatric solid malignancy, representing approximately 8% of all childhood cancers [1]. Despite advancements in treatment, the prognosis for high-risk patients remains unfavorable [2]. Although the oncogene MYCN plays a crucial role in neuroblastoma and is associated with poor outcomes, amplification is observed in less than 25% of tumors [3]. This emphasizes the need to identify new biomarkers that can serve as therapeutic targets.

Cell adhesion molecules (CAMs) are membrane glycoproteins that play a significant role in cell migration, proliferation, and differentiation [4,5]. Contactin-1 (CNTN1) is a CAM primarily found on neurons and glial cells in the central nervous system, which is physiologically involved in axonal growth and neuron development and differentiation [6]. CNTN1 has also been associated with advanced tumor stage, regional and metastatic disease, and reduced survival in multiple malignancies [7,8,9,10]. CNTN1 promotes invasion, migration, and metastasis through the epithelial–mesenchymal transition (EMT) [11]. In prostate cancer, CNTN1 downregulates E-cadherin, leading to enhanced invasion and xenograft tumor formation. Moreover, in thyroid cancer, knockdown of CNTN1 decreases tumor cell proliferation and invasiveness by reducing cyclin D1 expression [9,10].

In this study, we analyzed publicly available datasets and identified CNTN1 as one of the most significantly overexpressed genes in neuroblastoma tumors. Interestingly, CNTN1 expression was associated with improved outcomes and inversely correlated with MYCN amplification, suggesting a potential protective role. However, the precise role of CNTN1 in neuroblastoma remains incompletely understood, and its potential as a therapeutic target remains largely unexplored. Therefore, we investigated the function of CNTN1 in neuroblastoma, specifically focusing on tumor growth and metastasis in xenograft mouse models. Our findings provide valuable insight into the role of CNTN1 in neuroblastoma and its potential as a novel therapeutic biomarker.

## 2. Materials and Methods

### 2.1. Cell Lines and Plasmids

The human neuroblastoma cell lines SK-N-AS (CRL-2137) and SK-N-DZ (CRL-2149) were obtained from ATCC and cultured in a humidified incubator at 37 °C and 5% CO_2_ in Dulbecco’s Modified Eagle’s Medium (DMEM) with 4.5 g/L glucose, L-glutamine, sodium pyruvate, 10% (*v*/*v*) heat-inactivated FBS (Sigma-Aldrich F2442, St. Louis, MO, USA), and 1% antibiotic-antimycotic solution (Corning MT300004CI, Manassas, VA, USA). Cells were transduced with pCDH1-SV40-Luc2 construct and selected with hygromycin (200 µg/mL) to generate luciferase reporters. Three single guide RNAs (gRNA) targeting human CNTN1 (5′-AAGAGAATATTCACTACCAG-3′, 5′-AGCATCTAATAACTACGGGA-3′, and 5′-CTGTTCCGGATATCCGATGG-3′) were sub-cloned into pLenti-CRISPR-V2 (Addgene plasmid #52961, Cambridge, MA, USA) to knockout CNTN1. Pooled knockout cells were selected using puromycin selection (1 µg/mL) and screened for gene disruption using immunoblotting. All cell lines were periodically authenticated through morphological inspection and mycoplasma testing.

### 2.2. Immunoblotting

To perform immunoblotting, cells were lysed in RIPA lysis buffer containing protease inhibitors. Protein concentration was determined using a Pierce BCA Protein Assay Kit (VWR Scientific PI23225, Rockford, IL, USA). The following primary antibodies were used for immunoblotting: contactin-1 (R&D Systems AF904, Minneapolis, MN, USA) and β-actin (Sigma A5441, St. Louis, MO, USA). After incubating with the primary antibodies, membranes were incubated with fluorophore-conjugated secondary antibodies. Signals were detected using a LI-COR Odyssey CLx Imager (Lincoln, NE, USA).

### 2.3. Orthotopic Neuroblastoma Mouse Model

Animal studies were conducted following the guidelines established by the Institutional Animal Care and Use Committee (IACUC #PRAM201145989) at the Penn State College of Medicine (Hershey, PA, USA). Male and female 6–8-week-old immunodeficient NOD Rag Gamma (NRG, Jackson 007799, Bar Harbor, ME, USA) mice were used for all experiments. An orthotopic model of adrenal neuroblastoma was established by injecting 200,000 wildtype (WT) or CNTN1 knockout (crCNTN1) SK-N-AS or SK-N-DZ cell lines suspended in a 50:50 mixture of Matrigel basement membrane matrix (Corning CB-40234, Bedford, MA, USA) into the adrenal gland of mice anesthetized with inhaled isoflurane. A Visualsonics Vevo 2100 ultrasound (Visualsonics, Toronto, ON, Canada) was used for injections, as previously described [12,13]. Animals that failed to engraft tumor cells (lacking luciferase reporter signals) were excluded from the experiments and analyses. At the experimental endpoint, mice were euthanized, and tissues were harvested for ex vivo analysis.

### 2.4. Luciferase Tumor Imaging

Primary tumor growth was evaluated weekly using the IVIS Lumina III in vivo bioluminescent imaging system (IVIS). Mice were anesthetized with 2.5% isoflurane (IsoSol Isoflurane, USP, VEDCO, St. Joseph, MO, USA) and injected subcutaneously with a solution of 30 mg/mL D-luciferin in PBS at a dose of 5 μL/g body weight, 5 min before imaging. Photon flux was calculated by measuring the whole-body region of interest (ROI).

### 2.5. Statistical Analysis

Neuroblastoma gene expression data were obtained from the TARGET dataset. The expression data from Affymetrix Human Exon Array platform has 247 samples (after excluding two samples with missing information). The GSE62564 gene expression profile was downloaded from GEO (www.ncbi.nlm.nih.gov/geo/, accessed on 4 November 2024). This dataset was accessed on 15 August 2021, which includes 498 primary neuroblastoma patient samples and 19,320 expressed genes. There are 176 high-risk and 322 low-risk NB patients available. GraphPad Prism (Version 8.3, GraphPad Software, Inc., Boston, MA, USA) was used for statistical analysis. For single comparisons, we used two-tailed unpaired Student’s *t*-tests. For multiple comparisons, we used one-way ANOVA. Statistical significance was set at *p* < 0.05.

## 3. Results

### 3.1. Association Between CNTN1 Expression and Tumor Aggression in Neuroblastoma

**Figure 1:**
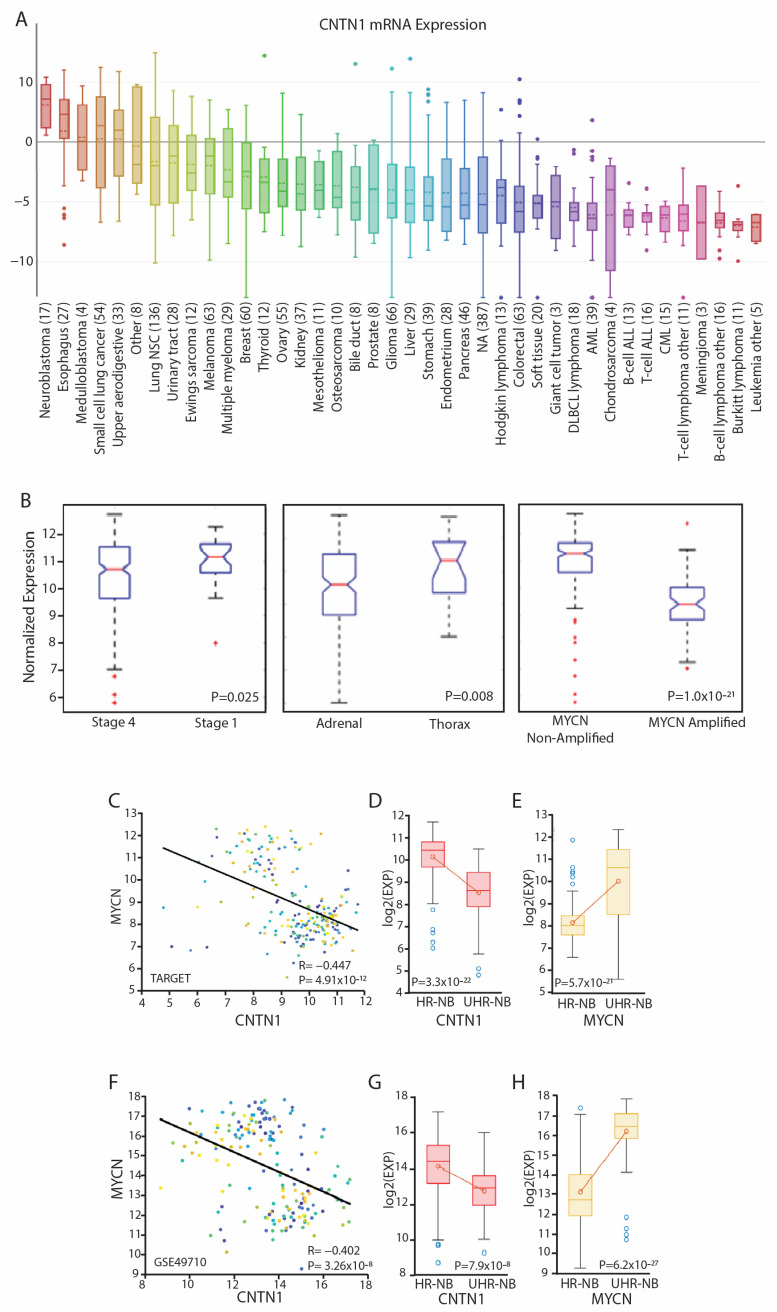
High CNTN1 expression is associated with less aggressive tumor characteristics in neuroblastoma patients. (**A**) CNTN1 mRNA expression from the Cancer Cell Line Encyclopedia (broadinstitute.org). Neuroblastoma, *n* = 17. (**B**) Normalized CNTN1 expression by primary tumor stage, location, and MYCN status. Stage 4, *n* = 216; stage 1, *n* = 30; adrenal, *n* = 149; thorax *n* = 19, non-MYCN-amplified *n* = 175; and MYCN-amplified *n* = 68. (**C**) CNTN1 expression compared to MYCN expression from the TARGET dataset, n = 217. (**D**,**E**) CNTN1 and MYCN expression in high-risk (HR) neuroblastoma samples stratified into HR and ultra-high risk (UHR) from the TARGET dataset, HR-NB *n* = 131, UHR-NB *n* = 86. (**F**) CNTN1 expression compared to MYCN expression from GSE62564 dataset, *n* = 176. (**G**,**H**) CNTN1 and MYCN expression in high-risk (HR) and ultra-high-risk (UHR) neuroblastoma from the GSE62564 dataset, HR-NB *n* = 102, UHR-NB *n* = 74.

To investigate the role of CNTN1 in neuroblastoma, we analyzed several large publicly available datasets of tumor gene expression. Our analysis of the Cancer Cell Line Encyclopedia (CCLE) revealed that CNTN1 mRNA expression was highest in neuroblastoma compared to other tumor types (Figure 1A), consistent with the known function of CNTN1 as an endogenous neuronal membrane glycoprotein.

The primary tumor site has been shown to correlate with patient prognosis in neuroblastoma. Adrenal-gland tumors are typically more aggressive and associated with poor outcomes, while tumors arising from other sites, such as the paraspinal ganglia, posterior mediastinum, abdomen, and pelvis, are associated with improved survival [14]. Analysis of the TARGET database revealed that CNTN1 expression was higher in tumors originating from the thorax compared to those from the adrenal gland (Figure 1D), in line with a potential protective role of CNTN1 in neuroblastoma. Due to low sample numbers, a statistical analysis was not feasible for tumors from the cervical, pelvic, extra-adrenal abdomen, and metastatic sites, as they constituted less than 10% of the overall dataset.

Further analysis of both the TARGET and GSE49710 demonstrated that CNTN1 expression was significantly higher in less aggressive stage 1 and non-MYCN-amplified tumors compared to more aggressive stage 4 and MYCN-amplified tumors, respectively (Figure 1D). In both datasets, there was a significant negative linear relationship between CNTN1 expression and MYCN, with high CNTN1 expression correlating with low MYCN expression (Figure 1E–H). When high-risk neuroblastoma patients were stratified into two clinically distinct subtypes, high-risk neuroblastoma (HR-NB) and ultra-high-risk neuroblastoma (UHR-NB), as described previously [15], we observed that CNTN1 expression was significantly higher in HR-NB tumors compared to UHR-NB tumors, while MYCN expression was significantly higher in UHR-NB tumors compared to HR-NB tumors in both datasets (Figure 1F,H).

### 3.2. Association Between CNTN1 Expression and Outcomes in Neuroblastoma Patients

**Figure 2:**
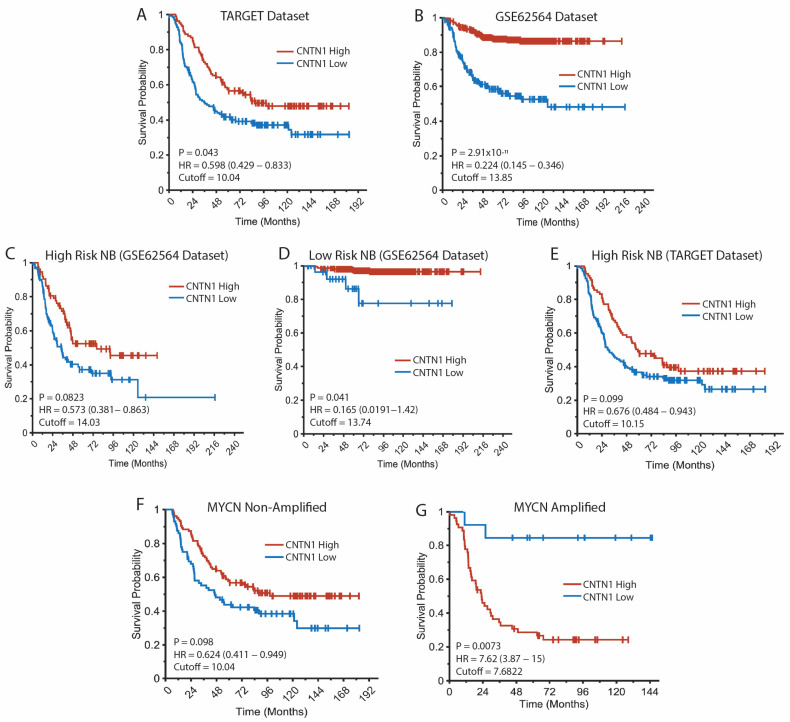
High CNTN1 expression is associated with improved outcomes in neuroblastoma patients. (**A**) Kaplan–Meyer overall survival curve of neuroblastoma patients from the TARGET dataset, stratified by CNTN1 expression with a cutoff of 10.04. (**B**) Kaplan–Meyer overall survival curve of neuroblastoma patients from the GSE62564 dataset, stratified by CNTN1 expression with a cutoff of 13.85. (**C**) Kaplan–Meyer overall survival curve of high-risk neuroblastoma patients from the GSE62564 dataset, stratified by CNTN1 expression with a cutoff of 14.03. (**D**) Kaplan–Meyer overall survival curve of low-risk neuroblastoma patients from the GSE62564 dataset, stratified by CNTN1 expression with a cutoff of 13.74. (**E**) Kaplan–Meyer overall survival curve of high-risk neuroblastoma patients from the TARGET dataset, stratified by CNTN1 expression with a cutoff of 10.15. (**F**) Survival ratio of neuroblastoma patients with non-MYCN-amplified tumors, stratified by CNTN1 expression with a cutoff of 10.04. (**G**) Survival ratio of neuroblastoma patients with MYCN-amplified tumors, stratified by CNTN1 expression with a cutoff of 7.6822.

To assess the prognostic role of CNTN1 in neuroblastoma, we analyzed the correlation between patient overall survival (OS) and CNTN1 gene expression using the survminer package in R, with the cutoff point determined by the surv_cutpoint function. The adjusted *p*-value was calculated using the conditional Monte Carlo method in the maxstat package to correct for multiple testing. High CNTN1 expression was significantly associated with increased overall survival probability in neuroblastoma patients from both the TARGET and GSE62564 datasets (Figure 2A,B). We stratified high- and low-risk neuroblastoma into subtypes based on CNTN1 expression and generated Kaplan–Meier plots for these subtypes using the GSE62564 dataset (Figure 2C,D), and for high-risk neuroblastoma using the TARGET dataset (Figure 2E). Due to the small sample size, we did not create a plot for low-risk neuroblastoma in the TARGET dataset. Upregulation of CNTN1 was significantly associated with improved overall survival in both high- and low-risk neuroblastoma patients with the adjusted *p*-value below 0.1. These findings suggest a potential protective role for CNTN1. Interestingly, when considering MYCN amplification across the entire cohort, high CNTN1 expression was associated with a significantly increased overall survival probability for neuroblastoma patients with non-MYCN-amplified tumors (Figure 2F). However, in patients with MYCN-amplified tumors, high CNTN1 expression was associated with a decreased probability of survival, although this finding was based on a relatively small number of patients with tumors exhibiting both MYCN amplification and low CNTN1 expression (Figure 2G).

### 3.3. CNTN1 Knockout Suppresses Tumor Metastasis and Prolongs Mouse Survival

**Figure 3:**
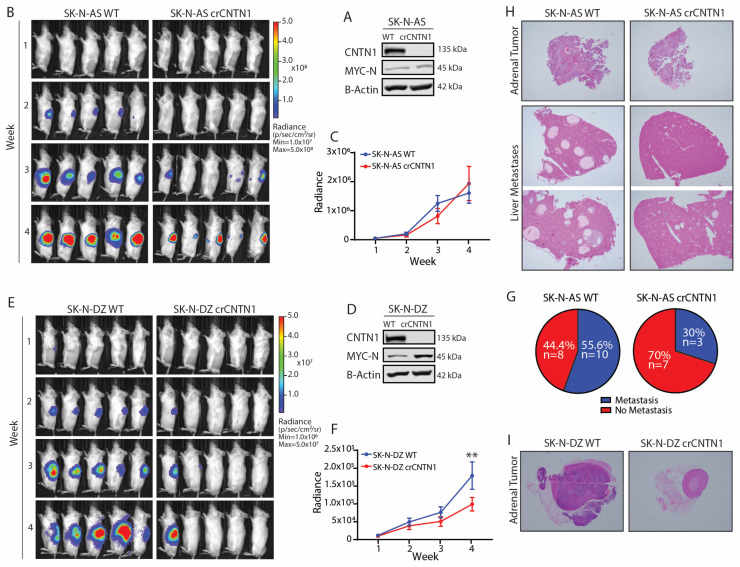
CNTN1 decreases survival and promotes tumor progression in vivo. (**A**) Immunoblot of WT and crCNTN1 SK-N-AS cells probed for indicated proteins. (**B**) Representative IVIS images of five mice xenografted with WT or crCNTN1 SK-N-AS tumors. (**C**) Whole-body ROI quantification of WT and crCNTN1 SK-N-AS tumor radiance. Data represent two independent experiments. SK-N-AS WT, *n* = 23; SK-N-AS crCNTN1, *n* = 11. Mean ± SEM. (**D**) Immunoblot of WT and crCNTN1 SK-N-DZ cells probed for indicated proteins. (**E**) Representative IVIS images of five mice xenografted with WT or crCNTN1 SK-N-DZ tumors. (**F**) Whole-body ROI quantification of WT and crCNTN1 SK-N-DZ tumor radiance. Data represent three independent experiments. SK-N-DZ WT, *n* = 37; SK-N-DZ crCNTN1, *n* = 31. Mean ± SEM, two-way ANOVA, ** *p* < 0.01. (**G**) Percentage of metastases identified in mice bearing WT or crCNTN1 SK-N-AS xenograft tumors. Data represent two independent experiments. SK-N-AS WT, *n* = 18; SK-N-AS crCNTN1, *n* = 10. (**H**,**I**) Representative hematoxylin–eosin-stained images of paraffin-embedded primary and metastatic tumors isolated from mice inoculated with indicated cell lines.

To investigate the effect of CNTN1 on primary neuroblastoma tumor growth and metastasis in vivo, we established CNTN1 knockout (crCNTN1) using a CRISPR-Cas9 system in both the non-MYCN-amplified SK-N-AS and the MYCN-amplified SK-N-DZ high-risk human neuroblastoma cell lines (Figure 3A,D). An orthotopic xenograft model of neuroblastoma was established by implanting either crCNTN1 or wildtype (WT) cells into the adrenal gland of NRG mice using ultrasound-guided imaging. We monitored tumor growth through weekly whole-body in vivo bioluminescent imaging (Figure 3B,E). There was no significant difference in primary tumor growth between the WT SK-N-AS and crCNTN1 SK-N-AS groups (Figure 3C). However, primary tumor growth significantly decreased in the crCNTN1 SK-N-DZ group compared to the WT SK-N-DZ group (Figure 3F). At the experimental endpoint, while no detectable metastasis was observed in mice with SK-N-DZ xenografts, we noted a higher frequency of visible metastatic tumor growth in mice with WT SK-N-AS tumors compared to those with crCNTN1 SK-N-AS tumors. Specifically, over 55% of mice in the WT group developed metastases, compared to 30% in the crCNTN1 group (Figure 3G). H&E staining of adrenal and liver tissues confirmed primary tumors in the adrenal glands and metastatic tumors in the liver (Figure 3H,I). These results suggest that CNTN1 loss suppresses metastasis in this mouse model.

## 4. Discussion

Neuroblastoma is a highly aggressive pediatric malignancy with relatively few therapeutic targets. Here, we demonstrate that neuroblastoma tumors express significantly higher levels of the neural glycoprotein CNTN1 compared to many other tumor types. Moreover, CNTN1 expression varies with tumor grade and primary tumor site, with higher expression observed in less aggressive grade 1 tumors, as well as tumors originating from the thorax, a clinically favorable primary site, compared to those in the adrenal gland. Importantly, high CNTN1 expression is associated with an increased overall survival probability in neuroblastoma patients.

Previous studies have demonstrated that CNTN1 is associated with poor overall survival in many tumor types [16]. Interestingly, low-grade glioma demonstrates the reverse phenotype, similar to our findings in neuroblastoma, where high CNTN1 is associated with increased overall survival [16]. Given the known role of CNTN1 in neural cell adhesion and axonal growth, these results suggest that CNTN1 could play an overall protective role in the progression of tumors of the nervous system, while promoting tumor progression through epithelial-to-mesenchymal transition (EMT) and other well-defined mechanisms in other tissue types.

Our study further reveals that patient survival based on CNTN1 expression is stratified by MYCN amplification status. High CNTN1 expression is associated with increased overall survival probability in patients with non-MYCN-amplified tumors, while high CNTN1 expression is linked to a decreased survival probability in patients with MYCN-amplified tumors. However, the sample size of patients with MYCN amplification and low CNTN1 expression is small. As there is no overlap between the CNTN1 and MYCN pathways to suggest that these proteins function synergistically, this finding may highlight the aggressive nature of MYCN-amplified tumors, but further experimental evaluation is needed to characterize this relationship.

Interestingly, in an orthotopic xenograft mouse model of neuroblastoma, we observed decreased metastasis in mice xenografted with CNTN1 knockout SK-N-AS tumors compared to mice with WT tumors. This finding is consistent with the literature on other tumor types demonstrating that CNTN1 promotes tumor metastasis [7,8,9,10,16], but it does not support the hypothesis that CNTN1 plays an overall protective role in neuroblastoma. For example, knockdown of CNTN1 has been shown to reduce the invasion of lung cancer [17], oral squamous cell carcinoma [18], and thyroid cancer cells [10], while overexpression increased lung metastasis in a mouse model of prostate cancer [9]. CNTN1 has been shown to promote tumor invasion primarily through the regulation of EMT [17,19,20,21], as well as the regulation of vascular endothelial growth factor (VEGF) [22,23,24,25].

It is important to note that this study has limitations, including the use of cell lines derived from the bone marrow of children with high-risk neuroblastoma, which were implanted into immunodeficient NRG mice. The effect of the tumor microenvironment and immune system on tumor progression cannot be accurately replicated using this experimental model. Additionally, the patients in the publicly available datasets used in this study underwent intensive treatments, including chemotherapy, surgery, stem-cell transplant, and/or immunotherapy, which is not reflected in the experimental animal model. Given differences in prior treatment for individual patients, the effect of CNTN1 on overall survival is most likely multifaceted.

## 5. Conclusions

Our findings highlight the potential significance of CNTN1 as a novel biological marker and therapeutic target for neuroblastoma. While patient data suggest that high CNTN1 expression is associated with less aggressive tumor characteristics and increased overall survival, animal studies suggest that CNTN1 knockout suppresses metastasis. Further studies are required to elucidate the mechanisms through which CNTN1 affects tumor growth and progression in neuroblastoma in order to evaluate its potential as a therapeutic target in preclinical and clinical settings.

## Data Availability

Data are contained within the article.
